# The potential of pale flax as a source of useful genetic variation for cultivated flax revealed through molecular diversity and association analyses

**DOI:** 10.1007/s11032-014-0165-5

**Published:** 2014-08-12

**Authors:** Braulio J. Soto-Cerda, Axel Diederichsen, Scott Duguid, Helen Booker, Gordon Rowland, Sylvie Cloutier

**Affiliations:** 1 Department of Plant Science, University of Manitoba, 66 Dafoe Road, Winnipeg, MB R3T 2N2, Canada; 2 Cereal Research Centre, Agriculture and Agri-Food Canada, 195 Dafoe Rd, Winnipeg, MB R3T 2M9 Canada; 3 Plant Gene Resources of Canada, Agriculture and Agri-Food Canada, 107 Science Place, Saskatoon, SK S7N 0X2 Canada; 4 Morden Research Station, Agriculture and Agri-Food Canada, 101 Route 100, Unit 100, Morden, MB R6M 1Y5 Canada; 5 Department of Plant Sciences, College of Agriculture and Bioresources, University of Saskatchewan, 51 Campus Drive, Saskatoon, SK S7N 5A8 Canada; 6 Present Address: Agriaquaculture Nutritional Genomic Center, CGNA, Genomics and Bioinformatics Unit, 4791057 Temuco, Chile; 7 Present Address: Eastern Cereal and Oilseed Research Centre, Agriculture and Agri-Food Canada, 960 Carling Avenue, Ottawa, ON K1A 0C6 Canada

**Keywords:** Pale flax, Cultivated flax, Genetic diversity, Association mapping, Wild ancestor, Exotic alleles

## Abstract

Pale flax (*Linum bienne* Mill.) is the wild progenitor of cultivated flax (*Linum usitatissimum* L.) and represents the primary gene pool to broaden its genetic base. Here, a collection of 125 pale flax accessions and the Canadian flax core collection of 407 accessions were genotyped using 112 genome-wide simple sequence repeat markers and phenotyped for nine traits with the aim of conducting population structure, molecular diversity and association mapping analyses. The combined population structure analysis identified two well-supported major groups corresponding to pale and cultivated flax. The *L. usitatissimum* convar. *crepitans* accessions most closely resembled its wild progenitor, both having dehiscent capsules. The unbiased Nei’s genetic distance (0.65) confirmed the strong genetic differentiation between cultivated and pale flax. Similar levels of genetic diversity were observed in both species, albeit 430 (48 %) of pale flax alleles were unique, in agreement with their high genetic differentiation. Significant associations were identified for seven and four traits in pale and cultivated flax, respectively. Favorable alleles with potentially positive effect to improve yield through yield components were identified in pale flax. The allelic frequencies of markers associated with domestication-related traits such as capsular dehiscence indicated directional selection with the most common alleles in pale flax being absent or rare in cultivated flax and vice versa. Our results demonstrated that pale flax is a potential source of novel variation to improve multiple traits in cultivated flax and that association mapping is a suitable approach to screening pale flax germplasm to identify favorable quantitative trait locus alleles.

**Electronic supplementary material:**

The online version of this article (doi:10.1007/s11032-014-0165-5) contains supplementary material, which is available to authorized users.

## Introduction

Plant breeders select favorable alleles to introduce new variation (Sim et al. 2011). Conversely, as the genetic base of breeding populations narrows through selection and fixation of specific alleles, breeding progress is hampered (Sim et al. 2011). Shrinking of the genetic diversity consequently reduces options to ensure diverse nutrition, to enhance food production and to face climate change. Crop wild relatives hosted in gene bank collections have received some attention because they harbor untapped genetic variation available for domesticated crops (McCouch et al. 2013; Xiao et al. 1998).

Pale flax (*Linum bienne* Mill.) is the wild progenitor of cultivated flax (*Linum usitatissimum* L.) as supported by morphological, cytological and molecular characterizations (Diederichsen and Hammer 1995; Fu and Allaby 2010; Fu and Peterson 2010; Gill and Yermanos 1967; Tammes 1928; Uysal et al. 2010, 2012). Pale flax is a winter annual or perennial plant with narrow leaves and dehiscent capsules and, usually displays large variation in the vegetative plant parts and variable growth habit (Diederichsen and Hammer 1995; Uysal et al. 2010, 2012). On the other hand, cultivated flax is mostly a spring annual plant that has variable seed dormancy, grows fast, flowers early, with almost indehiscent capsules and large seeds (Diederichsen and Richards 2003). Because both species share the same chromosome number (*n* = 15) and produce fully fertile offspring, pale flax represents cultivated flax’s primary gene pool (Diederichsen 2007), which can be accessed to broaden its generally narrow genetic bases (Cloutier et al. 2009; Diederichsen and Fu 2006; Fu et al. 2002, 2003; Smýkal et al. 2011; Soto-Cerda et al. 2012). However, there are no studies to date reporting the deployment of pale flax into cultivated flax breeding (Diederichsen 2007). In total, 279 pale flax accessions have been stored in gene banks around the world, the largest collection being at the German Institute of Plant Genetics and Crop Plant Research (IPK) (Diederichsen 2007). Recent efforts have expanded the pale flax germplasm to add 34 accessions collected in Turkey (Uysal et al. 2010).

Molecular markers have facilitated the study and comparison of the genetic diversity between cultivated crops and their wild relatives. Molecular diversity studies conducted between soybean (*Glycine max*) and *Glycine soja* (Lam et al. 2010), barley (*Hordeum* *vulgare*) and *Hordeum vulgare* ssp. s*pontaneum* (Russell et al. 2011), maize (*Zea mays*) and *Zea mays* ssp. *parviglumis* (Hufford et al. 2012), and rice (*Oryza sativa*) and *Oryza rufipogon* and *Oriza nivara* (Xu et al. 2013) have provided insights into the amount, distribution and erosion of the genetic variation present in cultivated crops relative to their wild ancestors. Nevertheless, these studies have mostly revealed patterns of variation at neutral loci, including allelic diversity, population structure and shared ancestry. Combined analysis of neutral and fitness-related loci between cultivated crops and wild relatives can assist in the identification and deployment of novel or eroded variation. Tanksley and Nelson (1996) and Zamir (2001) proposed the advanced backcross quantitative trait loci (AB-QTLs) and the introgression lines (ILs) methods, respectively, for the simultaneous detection of QTL and the development of improved varieties (Xiao et al. 1998). Although these approaches have proven successful for several crops (Liu et al. 2006; Schmalenbach et al. 2009; Tanksley 1993; Xiao et al. 1998), they screen a limited number of alleles per locus because they are based on biparental populations. Alternatively, association mapping (AM) utilizes a population of individuals representing a higher level of allelic diversity that improves the probability of QTL detection and the mapping resolution (Flint-Garcia et al. 2003). AM has identified favorable exotic alleles in *Solanum* *lycopersicum* var. *cerasiforme* (Ranc et al. 2012), *Hordeum vulgare* ssp. *spontaneum* (Matus et al. 2003) and *Zea mays* ssp. *parviglumis* (Weber et al. 2009).

In the present study, we conducted population structure, molecular diversity and AM analyses for 125 pale flax accessions and 407 cultivated flax accessions from the Canadian flax core collection (Diederichsen et al. 2013). The aims of our study were: (1) to understand the population structure and genetic relationships between pale and cultivated flax (2) to quantify their genetic diversity and (3) to identify QTL alleles in pale flax that could be used to broaden the allelic richness of cultivated flax for nine agronomic, phenological and morphological traits.

## Materials and methods

### Plant material, genotyping and field trials

One hundred and twenty-five pale flax accessions were provided by the Institute of Plant Genetics and Crop Plant Research (IPK), Plant Gene Resources of Canada (PGRC) and the US Department of Agriculture (USDA) (Online Resource 1). Among the pale flax accessions, those recently collected from Turkey were not included (Uysal et al. 2010). The Canadian flax core collection contains 381 accessions selected by Diederichsen et al. (2013), and twenty-six accessions of relevance to recent Canadian flax breeding programs. Both collections were genotyped with 112 simple sequence repeat (SSR) markers distributed across the 15 linkage groups of flax (Cloutier et al. 2012) using a single plant for each accession. The 112 SSRs were selected based on their polymorphic information content (PIC). The amplification products were resolved on an ABI 3130xl Genetic Analyzer (Applied Biosystems, Foster City, CA, USA). Output files were analyzed by GeneScan (Applied Biosystems) and subsequently imported into Genographer. Fragment sizes were estimated using GeneScan ROX-500 (Applied Biosystems) and MapMarker^®^ 1000 (BioVentures Inc., Murfreesboro, TN) internal size standards. The genotype of each locus was encoded based on its allele size in bp or as a null allele for dominant markers.

For pale flax, sixty-five accessions (Diederichsen and Hammer 1995) were phenotypically evaluated between 1983 and 1992. Every accession was cultivated for three years with two observations of ten plants every year (Diederichsen and Hammer 1995). For cultivated flax, 390 accessions from the Canadian flax core collection were evaluated during 4 years (2009, 2010, 2011 and 2012) at the Morden Research Station, Morden, Manitoba and at the Kernen Crop Research Farm located near Saskatoon, Saskatchewan, Canada. A type 2 modified augmented design (MAD) (Lin and Poushinsky 1985) was used for the field experiments from which phenotypic data were collected for nine traits. Main plots were arranged in grids of ten rows and ten columns. Each main plot was divided into five paralleled subplots (2 m × 2 m with 20 cm between rows) with a plot control (CDC Bethune) located in the center. Additional subplot controls (Hanley and Macbeth) were assigned to five randomly selected main plots.

### Phenotypic data

For pale flax, phenotypic data for thousand seed weight (TSW), seeds per boll (SPB), plant height (PH), capsular dehiscence (DEH), start of flowering (FL5%), petal color (PC), petal overlap (PO), flower shape (FS) and seed color (SC) were as described in Diederichsen and Hammer (1995). For cultivated flax, TSW and SPB were obtained by harvesting two half meter sections from the central part of each subplot. PH (cm) was recorded at maturity using the average of ten plants located in the center of the subplots. DEH was scored as follows: 1 = dehiscent, 2 = medium opened, 5 = slightly opened, 7 = weak, 9 = indehiscent. FL5 % was measured as the number of days between sowing and when 5 % of the flowers had opened. PC was scored using the following scale: 1 = white, 2 = light blue, 3 = blue, 4 = dark blue, 5 = pink, 6 = violet. PO was measured as follows: 1 = petal overlap more than 50 % of length, 2 = petal overlap less than 50 % of length. FS was recorded using the following scale: 1 = tube, 2 = funnel, 3 = bowl. SC was scored as follows: 1 = yellow, 2 = yellowish brown, 3 = olive, 4 = light brown, 5 = medium brown, 6 = dark brown.

### Statistical analysis

Pale flax data were statistically summarized (Diederichsen and Hammer 1995) and the mean for each trait was used for posterior analyses. For cultivated flax, adjusted data were obtained for each trait as previously described based on the MAD (You et al. 2013). Normal distribution of the mean adjusted traits was tested using the Shapiro–Wilk test (Shapiro and Wilk 1965). Traits with significant deviation from a normal distribution were log-transformed in pale and cultivated flax prior to AM analysis and that included PC, PO, FS and SC. For cultivated flax, an analysis of variance for the different sources of variation including year, location, genotype and their interactions was reported (Soto-Cerda et al. 2014).

### Population structure and phylogenetic analyses

To investigate the patterns of population structure, Bayesian-based and principal coordinate (PCoA) analyses based on the 112 SSRs including minor allele frequency (MAF) <0.05 were conducted. Population structure analysis was first carried out including both pale and cultivated flax accessions using STRUCTURE 2.3.3 (Pritchard et al. 2000). The admixture model with correlated allele frequencies was used with a burn in of 10,000 and 100,000 iterations for *K* populations ranging from 1 to 6. Population structure analysis was conducted separately for *K* populations ranging from 1 to 7. Ten runs for each *K* value were performed, and the ad hoc statistic ∆*k* was used to determine the optimum number of populations (Evanno et al. 2005). The membership coefficient estimate (*Q*) for each accession was calculated by averaging the ten runs of the best *K*. The inferred populations were merged post-analysis and visualized in Distruct (Rosenberg 2004). PCoA was performed in a multidimensional space with data standardization using GENALEX version 6.51 (Peakall and Smouse 2012). In addition, the 407 cultivated flax accessions were divided in four convarieties including *L. usitatissimum* convar. *mediterraneum* (15), *L. usitatissimum* convar. *usitatissimum* (304), *L. usitatissimum* convar. *elongatum* (83) and *L. usitatissimum* convar. *crepitans* (5), with the aim of identifying which convariety was the most closely related to pale flax. Pairwise *F*_ST_ comparisons and the unbiased Nei’s genetic distance parameter were calculated using GENALEX version 6.51 (Peakall and Smouse 2012) to determine the genetic differentiation between pale and cultivated flax. The partitioning of the genetic variation across populations identified by STRUCTURE was also performed using AMOVA, and the significance of each variation was estimated using 1,000 permutations in GENALEX version 6.51 (Peakall and Smouse 2012). Furthermore, the genetic differentiation among these populations was calculated as the pairwise *Φ*_ST_. To assess the genetic relationships between the wild progenitor and its cultivated form, a dendrogram was generated using the neighbor-joining (NJ) algorithm based on the Nei (1973) minimum genetic distance method implemented in PowerMarker version 3.25 (Liu and Muse 2005) and displayed by MEGA 5 (Tamura et al. 2011).

### Genetic diversity, relatedness and gain/loss of diversity

Genetic diversity parameters were estimated for the 125 pale flax and 407 cultivated flax accessions, and across the populations identified by STRUCTURE. The unbiased gene diversity (UH_e_), observed heterozygosity (*H*_o_), total number of alleles (*N*_a_), inbreeding coefficient (*F*_IS_) and polymorphic loci (%) were calculated in GENALEX version 6.51 (Peakall and Smouse 2012). Allelic richness (*R*_s_) and private alleles (*∏*) were corrected for sample size differences and estimated using the rarefaction method implemented in HP-RARE version 1.2 (Kalinowski 2005). The number of rare alleles (MAF < 0.05) and the PIC values were calculated in PowerMarker version 3.25 (Liu and Muse 2005). Significant differences between pale flax and cultivated flax genetic diversity parameters were estimated with the Kruskal–Wallis nonparametric test (Kruskal and Wallis 1952).

Relatedness was estimated using the molecular coancestry parameter (*f*_*ij*_) according to Caballero and Toro (2002). The molecular coancestry between two individuals *i* and *j* is the probability that two randomly sampled alleles from the same locus in two individuals are identical by state (Caballero and Toro 2002). Molecular coancestry matrices comparing all pairs of individuals within the pale and the cultivated flax collections, as well as within the populations identified by STRUCTURE were calculated using all 112 SSRs including MAF < 0.05 in MolKin version 3.0 (Gutiérrez et al. 2005).

The gain/loss of genetic diversity from the entire data set (532 accessions) when each population is removed was assessed with replacement of each population identified by STRUCTURE using the molecular kinship analysis proposed by Caballero and Toro (2002) implemented in MolKin version 3.0 (Gutiérrez et al. 2005). Caballero and Toro (2002) proposed setting priorities for conservation using the maintenance of the maximum overall Nei’s (1987) gene diversity (GD) in the preserved set of individuals. This is equivalent to the minimization of the overall molecular coancestry (*f*) because GD = 1 − *f* mean.

### Linkage disequilibrium in pale flax

In our previous study, we reported an average genome-wide LD (*r* ^2^) of 0.036, with a relatively fast decay of ~1 cM in the Canadian flax core collection (Soto-Cerda et al. 2013). In pale flax, LD was estimated based on the 65 accessions with available phenotypic data and used for AM by calculating *r* ^2^ in GGT 2.0 (van Berloo 2008). The absence of a pale flax linkage map imposed the use of the surrogate cultivated flax linkage map. This assumption is based on the morphological, cytological, molecular characterizations and SSR markers transferability between both species (Diederichsen and Hammer 1995;; Fu and Allaby 2010; Fu and Peterson 2010; Gill and Yermanos 1967; Soto-Cerda et al. 2011; Tammes 1928; Uysal et al. 2010, 2012). Allele’s frequency was calculated in PowerMarker version 3.25 (Liu and Muse 2005), and MAF < 0.05 was set to “U” (missing data) and excluded from the LD analysis. Genetic distances between markers were obtained from the SSR consensus linkage map of flax (Cloutier et al. 2012) integrated with the physical map (Ragupathy et al. 2011). In total, 104 SSRs with known chromosome information in the consensus map of flax (Cloutier et al. 2012) were used for LD estimation. SSRs on the same linkage group were considered linked and those on different linkage groups, unlinked. All unlinked LD pairwise comparisons were arbitrarily assigned to 140 cM, because at this distance none of the linked pairwise LD comparisons were located. Average genome-wide LD decay versus genetic distance was estimated as described (Breseghello and Sorrells 2006) and displayed in a LD scatter plot. A statistical cutoff value of *r* ^2^ = 0.1 was set to estimate the average genome-wide LD block.

### Association mapping

The mean phenotypic values and the mean of the adjusted phenotypic values of nine traits were used for AM in pale flax and cultivated flax, respectively. Five AM models were tested in TASSEL 2.1 (Bradbury et al. 2007) including two general linear models (GLMs) (*Q* and PCA) and three mixed linear models (MLMs) (*K*, *Q* + *K* and PCA + *K*) (Price et al. 2006; Pritchard et al. 2000; Yu et al. 2006). The *Q* matrix was estimated using the 112 SSRs in STRUCTURE. The PCA matrix calculated in TASSEL 2.1 retained the first three components. The *K* matrix was constructed on the basis of 112 SSRs using SPAGeDi (Hardy and Vekemans 2002). All negative values between individuals were set to zero (Yu et al. 2006). The best AM model was selected using probability–probability (*P*–*P*) plots. For the AM analysis, only minor allele frequency (MAF) > 0.05 was retained (Breseghello and Sorrells 2006).

Correction for multiple testing was performed using the estimated false discovery (*q*FDR) values (Benjamini and Hochberg 1995). The *q* values were calculated with the QVALUE R package using the smoother method (Storey and Tibshirani 2003). Markers with *q*FDR < 0.01 were considered significant. For markers significantly associated with a trait, a GLM with all fixed-effect terms was used to estimate the amount of phenotypic variation explained by each marker (*R* ^2^). Allelic effects of the significant marker loci were calculated as the difference between the average phenotypic values of the homozygous alleles with MAF > 0.05. The significant differences between the allele means were estimated by the Kruskal–Wallis nonparametric test (Kruskal and Wallis 1952) and visualized as box plots.

## Results

### Population structure and phylogenetic analyses

**Fig. 1:**
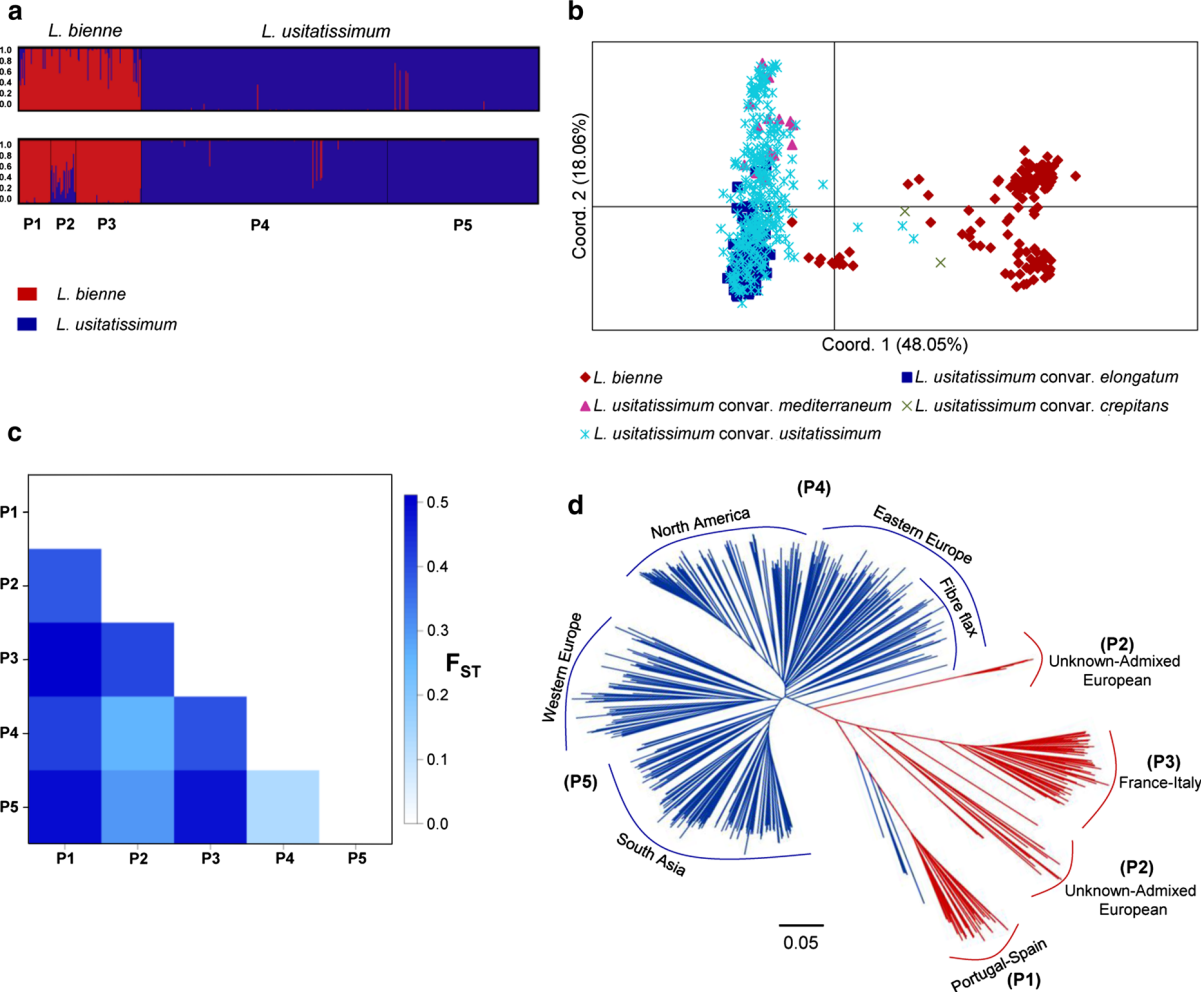
Population structure and genetic relationships between pale flax (*L. bienne*) and cultivated flax (*L. usitatissimum*). **a** structure analysis for *K* = 2 (*upper panel*) and the merged STRUCTURE (*K* = 2 and *K* = 3) populations (*lower panel*) of *L. bienne* (*red*) and *L. usitatissimum* (*blue*) accessions. P1, P2 and P3 represent populations within *L. bienne* major group and P4 and P5 represent populations within *L. usitatissimum* major group. **b** Principal coordinate analysis (PCoA) of the 532 pale and cultivated flax accessions. Cultivated flax was further divided into four convarieties, namely *L. usitatissimum* convar. *mediterraneum*, *L. usitatissimum* convar. *usitatissimum*, *L. usitatissimum* convar. *elongatum* and *L. usitatissimum* convar. *crepitans*. **c** Pairwise *F*_ST_ comparisons among the five populations inferred by STRUCTURE. **d** Phylogenetic tree created using the Neighbor-joining (NJ) algorithm (Nei 1973).* Colored* clusters represent pale and cultivated flax STRUCTURE (*K* = 2) major groups. P1–P5 correspond to the merged STRUCTURE (*K* = 2 and *K* = 3) populations, indicating their geographic distribution. The *scale bar* indicates the Nei (1973) minimum genetic distance

The Bayesian-based clustering approach implemented in STRUCTURE identified two major groups according to the ∆*k* approach (Fig. 1a, Online Resource 2), which corresponded to the pale and cultivated flax accessions. Separate analysis of the 125 pale flax accessions provided support for the existence of three populations named P1, P2 and P3 (Fig. 1a, Online Resource 2), which clustered accessions from Portugal–Spain, unknown-admixed European and France–Italy, respectively. P2 showed the largest proportion of shared alleles with cultivated flax. Separate analysis of the 407 cultivated flax accessions supported the existence of two major populations, here named P4 and P5 (Fig. 1a), in agreement with our previous study (Soto-Cerda et al. 2013). P4 contained mostly accessions from North America and Eastern Europe with a strong effect of the fiber morphotype from Eastern European countries, while P5 mostly clustered accessions from South Asia and Western Europe. Similar to the STRUCTURE analysis, the PCoA revealed the presence of two major groups where coordinates 1 and 2 explained 66 % of the total genetic variation (Fig. 1b). A few pale flax accessions from P2 grouped close to the fiber flax accessions, of which the majority corresponded to the convar. *elongatum* (Fig. 1a, b). Likewise, the convar. *crepitans* accessions clustered close to the pale flax major group, having in common dehiscent capsules (Fig. 1b).

The coefficient of population differentiation *F*_ST_ and the unbiased Nei’s genetic distance parameter between pale and cultivated flax was estimated at 0.23 (*P* < 0.001) and 0.65, respectively, indicating a strong population structure. Pairwise *F*_ST_ among the five populations identified by STRUCTURE ranged from 0.07 (*P* < 0.001) between P4 and P5 to 0.35 (*P* < 0.001) between P1 and P5 (Fig. 1c). Among the pale flax populations, P2 exhibited the weakest population structure when compared to the cultivated flax populations (Fig. 1c).

The AMOVA showed highly significant (*P* < 0.001) differentiation between the five STRUCTURE populations, which indicated that 79 % of the observed variation can be explained by the within populations subdivision (Online Resource 3). The *Φ*_ST_ value (0.21) indicated a strong population differentiation similar to the *F*_ST_ value. The high *F*_ST_, *Φ*_ST_ and unbiased Nei’s genetic distance values between pale flax and cultivated flax suggest that AM analysis should be conducted separately.

The phylogenetic analysis using the NJ algorithm also partitioned the 532 accessions into two major groups, largely corresponding to the pale and cultivated flax major groups identified by STRUCTURE and PCoA (Fig. 1d). Further, the cultivated flax accessions were divided into two populations resembling the P4 and P5 STRUCTURE populations. Likewise, the pale flax accessions were divided into three populations also resembling but not identical to the P1, P2 and P3 STRUCTURE populations (Fig. 1d). Similarly to the PCoA, a few pale flax accessions from P2 grouped close to the fiber flax cultivars, while the five convar. *crepitans* accessions clustered close to the pale flax accessions from Portugal–Spain.

### Genetic diversity, relatedness and gain/loss of diversity

In pale flax, the mean number of alleles adjusted for sample size (N_a_adj) was 8.03, of which, 55.6 % had a MAF < 0.05 and were considered rare alleles (*R*_a_) (Table 1). In comparison, the mean N_a_adj was 6.59 in the cultivated flax accessions, of which, 58.5 % were considered rare alleles. The observed heterozygosity (*H*_o_), the total unbiased gene diversity (UH_e_), the inbreeding coefficient (*F*_IS_), the allelic richness (*R*_s_), the number of private alleles (*∏*), the level of relatedness (*f*_*ij*_) and the gain/loss of genetic diversity parameters for the 125 pale flax accessions, the 407 cultivated flax accessions and the five STRUCTURE populations are summarized (Table 1). The cultivated flax accessions harbored similar genetic diversity to that observed in pale flax, based on the nonsignificant mean differences (*P* > 0.05) for *N*_a_, *H*_o_, *R*_s_ and *R*_a_ but for UH_e_ (*P* = 0.011) and ∏ (*P* = 1.41E−6). In general, P2 and P4 exhibited superior genetic diversity parameters within their respective species.

**Table 1:** Genetic diversity in pale flax and cultivated flax STRUCTURE populations

Population	*N* ^1^	*N*_a_^2^	*N*_a_adj^3^	*H*_o_^4^	UH_e_^5^	*F* ^6^	*R*_s_^7^	*Π* ^8^	*R*_a_^9^	*f*_*ij*_^10^	Gain (+)/loss (−) (%)^11^	Polymorphic loci (%)
Pale flax	125	899	899	0.02	0.59	0.97	7.96	430	500	0.33	−14.7	100
P1	32	387	376	0.01	0.37	0.95	3.36	93	100	0.33	−1.43	79.5
P2	26	529	529	0.02	0.55	0.98	4.67	110	178	0.24	−0.61	98.2
P3	67	541	437	0.02	0.35	0.93	3.90	97	285	0.33	−2.24	92.9
Cultivated flax	407	838	738	0.03	0.53	0.95	6.24	269	490	0.29	−4.37	100
P4	253	790	544	0.03	0.53	0.95	4.86	81	437	0.24	+0.68	99.1
P5	154	601	440	0.03	0.43	0.93	3.93	37	301	0.29	+1.67	99.1

^1^Number of accessions

^2^Number of alleles

^3^Number of alleles adjusted for sample size

^4^Observed heterozygosity

^5^Unbiased gene diversity

^6^Inbreeding coefficient

^7^Allelic richness

^8^Number of private alleles estimated on a sample of balanced size using rarefaction method (Kalinowski 2005)

^9^Rare alleles <5 %

^10^Molecular coancestry

^11^Gain/loss of genetic diversity (Caballero and Toro 2002)

The *f*_*ij*_ for pale flax was estimated at 0.33, whereas it was 0.29 for cultivated flax. The intrapopulation *f*_*ij*_ ranged from 0.24 (P2 and P4) to 0.33 (P1 and P3). The coancestry analysis indicated that most of the accessions within the five populations had weak familial relatedness.

The gain/loss of genetic diversity was assessed with replacement using the molecular kinship analysis. The greatest loss of genetic diversity was observed when the 125 pale flax accessions were removed from the data set (~15 %). Thereafter, the 125 pale flax accessions were added in the data set and the 407 cultivated flax accessions removed, generating ~4.4 % loss of genetic diversity (Table 1). When any of the pale flax populations were removed, there was a loss of diversity while removing any of the cultivated flax populations caused a gain of diversity. These results suggest that redundant alleles exist within P4 and P5, but unique allelic variation is present in pale flax populations, in agreement with the large number of private alleles.

### Linkage disequilibrium in pale flax

To analyze LD variation, genetic distances for 104 SSRs were available from the consensus linkage map of flax (Cloutier et al. 2012). In the 65 pale flax accessions assessed, the average *r* ^2^ values for linked and unlinked markers were 0.12 and 0.09, respectively. The average genome-wide LD decayed to 0.1 within 1.8 cM (Online Resource 4). Strong LD (*r* ^2^ > 0.4), suitable for efficient marker assisted selection (MAS), decayed even faster, to less than 0.1 cM.

### Association mapping

**Fig. 2:**
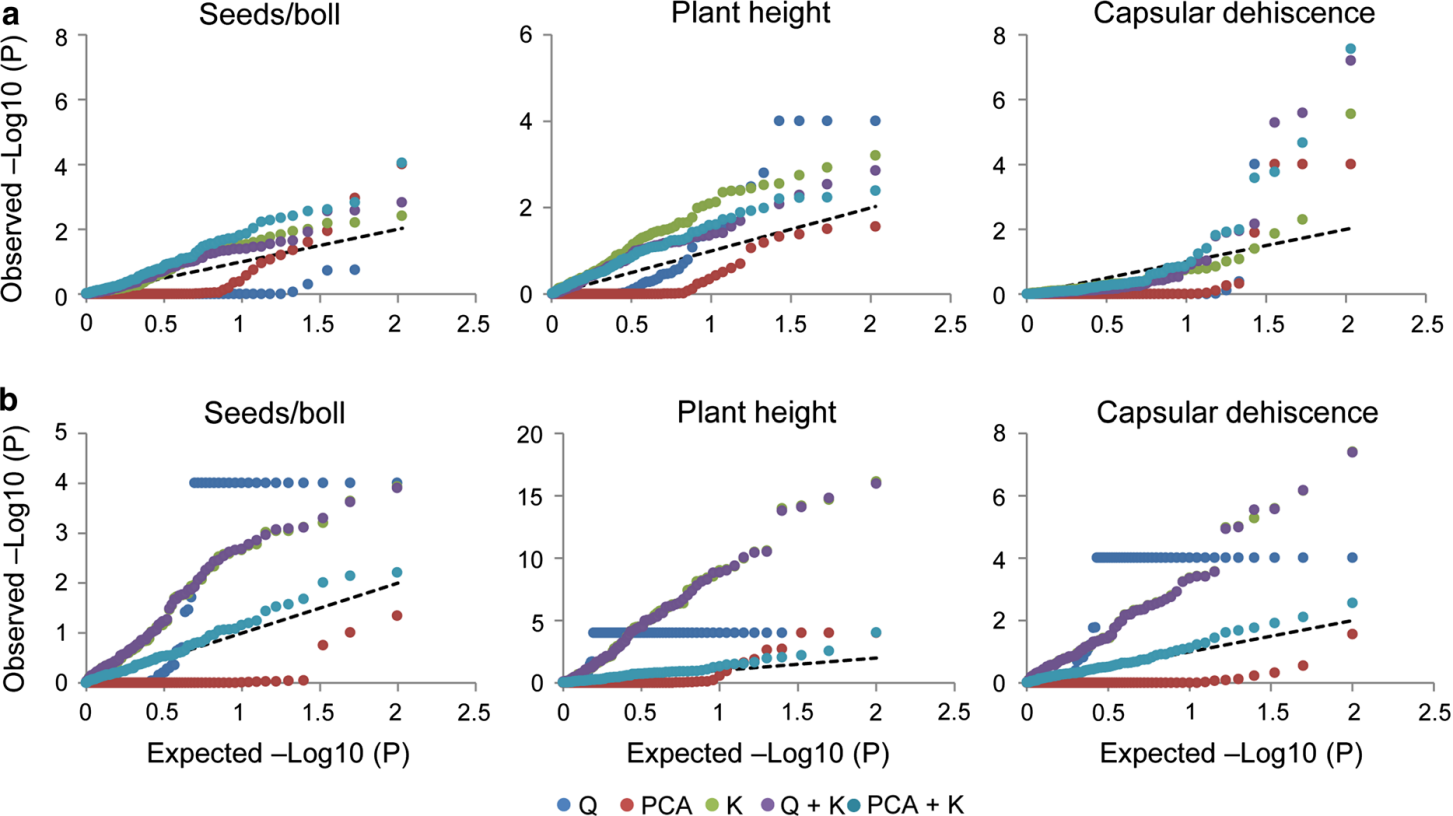
Comparisons of five association mapping models in pale and cultivated flax for seeds per boll, plant height and capsular dehiscence. Probability–probability (*P*–*P*) plots of observed versus expected −Log_10_ (*P*) values for **a** Pale flax. **b** Cultivated flax. *Q* general linear model using the *Q* matrix, PCA general linear model using the PCA matrix, *K* mixed linear model using the kinship matrix, *Q* + *K* mixed linear model using the *Q* and *K* matrices, PCA + *K* mixed linear model using the PCA and *K* matrices

As depicted by the *P*–*P* plots for both pale and cultivated flax, numerous spurious associations for all traits were observed with the GLM (*Q*) (Fig. 2, Online Resource 5). This model was characterized by an excess of small *P* values causing spurious associations. On the other hand, the GLM (PCA) over corrected the majority of the small *P* values. The MLMs performed differently in both species. For example, in pale flax, the *K* and *Q* + *K* models had the smallest deviations from the expected distribution for SPB and PH, while the PCA + *K* model was the most suitable for the other seven traits (Fig. 2a, Online Resource 5). In cultivated flax, the PCA + *K* model had the smallest deviation from the expected distribution for all traits (Fig. 2b, Online Resource 5). Taking into account the *P*–*P* plot results, three different MLMs were used for AM in pale flax, whereas only the PCA + *K* model was used in cultivated flax.

### Marker-trait associations

Association mapping was carried out to identify SSRs potentially associated with the nine traits. Significant associations (*q*FDR < 0.01) after Bonferroni correction (0.05/112 = 4.46E−4) were identified for seven and four of the nine traits in pale and cultivated flax, respectively. An overview of the associated markers, their map position, correlations with traits (*R* ^2^) and the effects of favorable alleles is presented in Table 2. A single marker for SPB, PH, FL5 % and PC and two for TSW and DEH were identified in pale flax, while a single marker was identified for TSW, PH and PO and two for FL5 % in cultivated flax but none were common between the two species. In pale flax, the percentage of the total phenotypic variation explained by the associated markers ranged from 1.3 to 97 %, whereas in cultivated flax it ranged from 1.4 to 16.3 % (Table 2).

**Table 2:** Marker loci significantly associated with thousand seed weight (TSW), seeds per boll (SPB), dehiscent capsules (DEH), plant height (PH), start of flowering (FL5 %), flower shape (FS), petal color (PC) and petal overlap (PO), and their explained phenotypic variance (*R* ^2^), the allelic effects of favorable alleles and their frequencies

Species	Trait	Marker	LG (cM)	*P* value	*R* ^2^ (%)	Effect^a^	Favorable allele (bp)	Frequency (%)
Pale flax	TSW	Lu451b	Unknown	1.42 E−4**	18.9	0.07 g	202	78.2
		Lu652	5 (79.4)	9.54 E−5**	28.8	0.49 g	274	9.2
	SPB	Lu1171	8 (27.6)	3.28 E−4**	4.8	1.56 U	280	5.8
	DEH	Lu2344	2 (89.3)	2.20 E−5**	20.1	0.1 U	303	47.1
		Lu442a	6 (44.3)	2.77 E−8**	9.0	0.08 U	267	14.4
	PH	Lu265	8 (37.1)	5.76 E−4*	14.3	−20.43 cm	null	52.3
	FL5%	Lu271	15 (53.6)	4.91 E−4*	8.5	−11.74 d	271	92.6
	FS	Lu2344	2 (89.3)	2.98 E−5**	1.31	0.60 U	315	15.9
	PC	Lu2725	10 (40.1)	2.00 E−13**	96.9	4 U	360	5.9
Cultivated flax	TSW	Lu2042	12 (18.2)	7.64 E−5**	6.74	1.46 g	162	7.0
	PH	Lu2067a	2 (59.7)	9.45 E−5**	16.3	−20.14 cm	205	27.6
	FL5%	Lu943	1 (150.0)	3.86 E−4**	9.2	−2.5 d	271	60.8
		Lu2067a	2 (59.7)	2.09 E−5**	2.3	−2.1 d	209	14.7
	PO	Lu3038	14 (32.2)	7.95 E−4*	1.4	0.1 U	303	6.2

** Significant at *q*FDR < 0.01 and after Bonferroni correction (0.05/112 = 4.46 E−4)

* Significant at *q*FDR < 0.01

^a^Effects of favorable alleles are represented in grams (g) for TSW, days (d) for FL5%, centimeters (cm) for PH and units (u) for SPB, DEH, FS, PC and PO

**Fig. 3:**
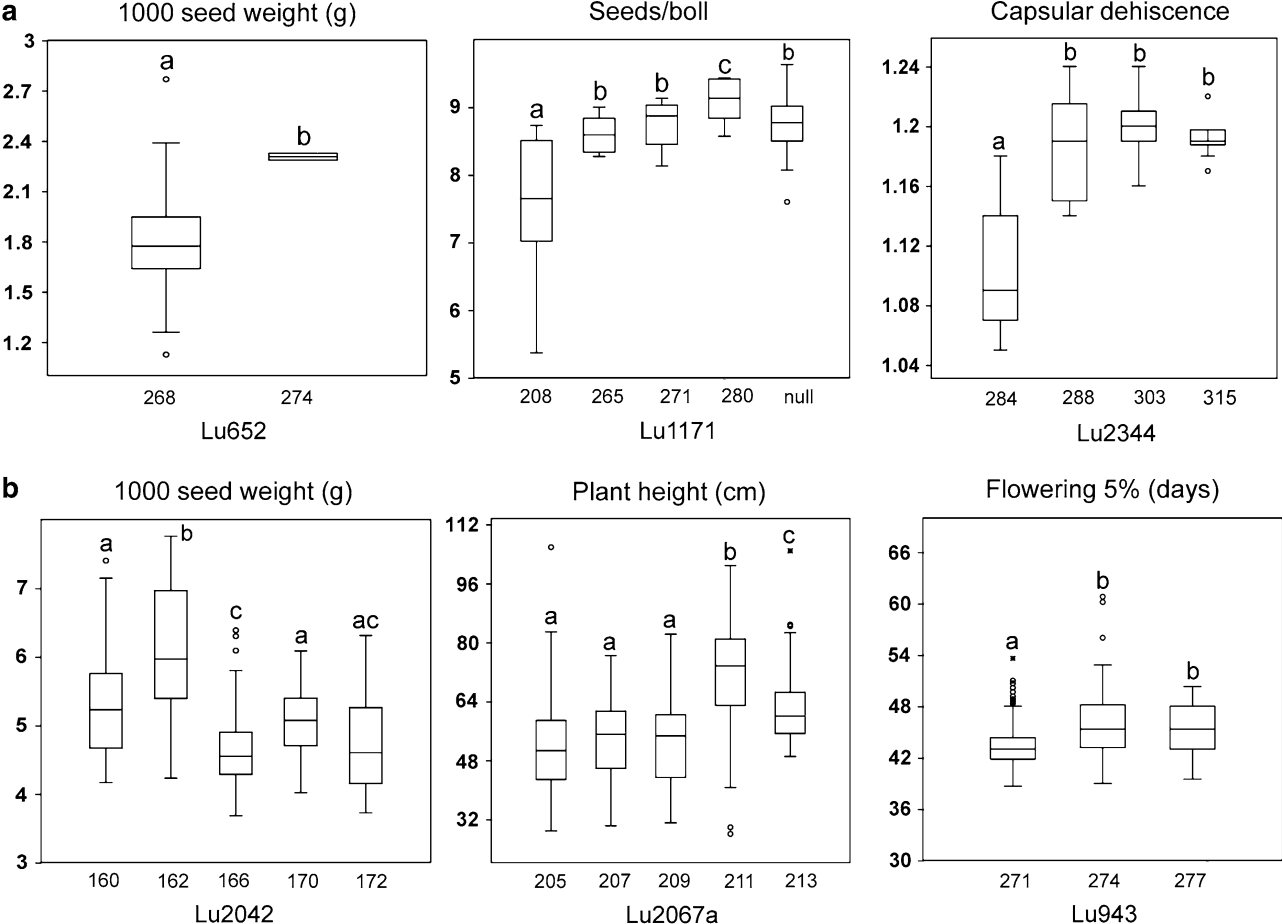
Comparisons of allelic effects of associated markers with agronomic and phenological traits. **a** Allelic effects on thousand seed weight, seeds per boll and capsular dehiscence in pale flax. **b** Allelic effects on thousand seed weight, plant height and 5 % flowering in cultivated flax. *Same letters above the box plots* indicated values that do not differ statistically according to the Kruskal–Wallis test (*α* = 0.01)

In pale flax, TSW could be significantly increased by an average of 0.49 g (*P* = 0.002) as an effect of the 274-bp allele of marker Lu652 (Fig. 3a). Similarly, the 280-bp allele of marker Lu1171 had the largest effect on SPB, increasing it by 1.56 seeds (*P* = 0.01). An increase of 0.1 units (*P* = 0.0012) in DEH was associated with the 303-bp allele of Lu2344, but all the genotypes carrying it had dehiscent capsules (Fig. 3a). For cultivated flax, TSW could be improved by an average of 1.64 g (*P* = 6.3E−4) as an effect of the 162-bp allele of marker Lu2042 (Fig. 3b). A reduction of up to 20.14 cm (*P* = 9.2E−4) in PH was associated with the 205-bp allele of Lu2067a compared with the 213-bp allele (Fig. 3b). The 271-bp allele of Lu943 significantly shortened FL5 % by 2.5 days (*P* = 3.65E−5) compared to the other two alleles (Fig. 3b).

### Allelic abundance and frequency of associated markers

The number of alleles harbored by the associated loci, including MAF < 0.05, was compared between pale and cultivated flax (Online Resource 6). Taking into account all the markers identified in both species, the observed number of alleles in pale flax was 110 (9.2/locus) while in cultivated flax it was 99 (8.2/locus). A total of 53 common alleles (4.4/locus) were observed across the twelve associated loci. In general, pale flax harbored more alleles than cultivated flax but as indicated by the Kruskall–Wallis test, the means were not statistically different (*P* = 0.321). For the twelve associated loci, pale flax registered 57 private alleles. The 262-, 265- and 277-bp alleles of marker Lu451b and the 208- and 247-bp alleles of marker Lu652 associated with TSW in pale flax were absent in cultivated flax (Online Resource 7). Similarly, marker Lu1171 associated with SPB registered six alleles absent in cultivated flax including the 280-bp allele with the largest effect on SPB (Table 2). For the same marker, however, the most frequent alleles in cultivated flax (265 and 268 bp) were rare or absent in pale flax (Online Resource 7). Marker Lu265 associated with PH in pale flax showed nine alleles absent in cultivated flax although at low frequencies (Online Resource 7). The most common alleles in cultivated flax, 256 and 259 bp (combined frequency of 90 %), were rare or absent in pale flax. Similarly, the null allele with the largest effect and highest frequency in pale flax was absent in cultivated flax (Table 2, Online Resource 7). For FL5 %, pale flax had one allele that was absent in cultivated flax (Online Resource 7).

Notably, among the 65 pale flax accessions, six had violet petals. All violet accessions carried the 360-bp allele of marker Lu2725 which increased PC by four units (Table 2). Conversely, alleles 330-, 332- and 334-bp alleles (combined frequency of 67 %), associated with light blue petals, were absent (332) and rare (330 and 334) in cultivated flax (Online Resource 7).

Likewise, the alleles of markers Lu2344 and Lu442a, associated with DEH in pale flax, were either absent or rare in cultivated flax. For example, the 284-, 288- and 315-bp alleles of Lu2344 were absent in cultivated flax, while the 285- and 303-bp alleles had frequencies of 1.1 and 3.7 %, respectively (Online Resource 7). The five (~1.1 %) convar. *crepitans* accessions characterized by their dehiscent capsules were the only ones carrying the 285-bp allele. In pale flax, however, the 284-, 285-, 288-, 303- and 315-bp alleles of Lu2344 had frequencies in the range of 4.1 to 45 %. Moreover, the two most common alleles in cultivated flax, the 297 (52.2 %) and 306 bp (33.5 %) were absent in the first case and rare in the second in pale flax. Because the low marker density might have influenced our ability to detect significant associations for DEH in cultivated flax, we considered 464 genome-wide SSRs from our previous work (Soto-Cerda et al. 2014) to conduct AM. Despite the increase marker density, no associations were detected, likely because of the small number of dehiscent accessions in the Canadian core collection (data not shown).

## Discussion

The remarkable diversity of wild relatives offers a reservoir of genetic variation that has the potential to positively impact crop improvement and support our food system in the future (McCouch et al. 2013). The genetic diversity in cultivated flax, one of the oldest oil and fiber crop domesticated by early civilizations, has been generally described as narrow (Cloutier et al. 2009; Diederichsen and Fu 2006; Fu et al. 2002, 2003; Smýkal et al. 2011; Soto-Cerda et al. 2012). Here, we reported on the genetic diversity, population structure and AM of the two largest collections of cultivated flax (Diederichsen and Fu 2006; Everaert et al. 2001; Fu et al. 2002, 2003; Fu 2005; Rajwade et al. 2010; Smỳkal et al. 2011; Soto-Cerda et al. 2012) and its wild progenitor, pale flax (Allaby et al. 2005; Diederichsen and Hammer 1995; Fu 2011; Uysal et al. 2010, 2012). Cultivated and pale flax possess similar levels of genetic diversity, and pale flax could be a source of useful unique alleles for economically important traits in cultivated flax.

### Population structure and phylogenetic analyses

The STRUCTURE, PCoA and phylogenetic analyses were fully consistent with the phenotypic differences exhibited by pale and cultivated flax (Diederichsen and Hammer 1995; Uysal et al. 2012), as both species were separated into two genetically distinct groups. Further, the PCoA and the NJ tree, showed that the dehiscent convar. *crepitans* accessions most closely resembled its wild progenitor.

The *F*_ST_ pairwise comparisons indicated stronger population structure between pale flax populations than between cultivated flax populations, a phenomenon that could be explained by limited gene flow or strong local adaptation of pale flax accessions (Uysal et al. 2010). Conversely, the weak *F*_ST_ value between cultivated flax populations might reflect the more intensive germplasm exchange between gene banks and breeding programs around the world (Maggioni et al. 2002). Overall, our combined population structure analyses were consistent with clustering patterns obtained in previous molecular and phenotypic studies (Fu 2011; Uysal et al. 2010, 2012), supporting capsular dehiscence as the primary trait modified in early flax domestication, preceding other domestication traits such as oil content and fiber length (Fu 2011; Uysal et al. 2010).

### Genetic diversity, relatedness and gain/loss of diversity

The universality of loss of diversity in cultivated crops relative to their wild ancestors is well documented (Dubkovsky and Dvorak 2007; Liu and Burke 2006; Wright and Gaut 2005). However, we observed a similar level of diversity for most of the parameters between pale and cultivated flax (Table 1). ISSR analysis revealed similar results despite the limited number of accessions (Uysal et al. 2010). These findings suggest that much of the diversity that survived through the stages of domestication has been retained in gene banks and is well represented in the Canadian flax core collection (Diederichsen et al. 2013). Nevertheless, these seemingly similar levels of diversity could be misleading because the pale flax group of only 125 accessions is not a core collection but represent a collection primarily selected based on the availability and accuracy of the passport data and not based on diversity parameters. As a consequence, differences in population size and sampling methods can inflate the diversity harbored by the flax core collection as compared to a random sample of cultivated flax, making our comparisons biased.

The significantly higher percentage of genetic variation contained within populations as indicated by AMOVA, the number of private alleles and the 15 % loss of diversity when the 125 pale flax accessions were removed from the entire data set support the widely held view that pale flax is a potential source of novel alleles for cultivated flax improvement and therefore deserves special conservation efforts (Diederichsen 2007). The weak intrapopulation relatedness suggests that intra- as well as interpopulation breeding could be fruitful.

### Linkage disequilibrium in pale flax

Generally, LD decays more slowly in selfed pollinated species as compared to outcrossing ones (Flint-Garcia et al. 2003). Further, in selfed pollinated species, LD declines more rapidly in wild relatives and landraces than in their cultivated counterpart such as *O. rufipogon* (Xu et al. 2013), *G. soja* (Lam et al. 2010) and *H. vulgare* ssp. *spontaneum* (Morrell et al. 2005). In pale flax, the average LD block based on 65 accessions was estimated at ~1.8 cM. However, it is expected that using the 125 accessions, LD should decays more rapidly to similar levels or even faster than the ~1 cM estimated in the Canadian flax core collection (Soto-Cerda et al. 2013) based on the assumption that the marker order in a linkage map of pale flax would be similar to that observed in the linkage map of cultivated flax. An extreme example where LD remains high over long genetic distance (6–20 cM) is cultivated tomato where several bottlenecks that occurred during domestication hamper the use of AM (van Berloo 2008). Nevertheless, part of the cultivated tomato germplasm displays a genetic admixture with the wild relative *S. lycopersicum* var. *cerasiforme*. Such admixture could be compared with a multi parent advanced generation intercross (MAGIC) population that would provide greater level of genetic diversity and faster LD decay (Ranc et al. 2012). Similarly, pale flax can be used not only as a source of novel alleles for flax breeding but also to design interspecific MAGIC populations harboring superior allelic diversity and smaller LD blocks, both desirable properties for AM.

### Association mapping

An AM panel of diverse breeding history and/or geographic origin might exhibit population and family structure, both recognized as sources of spurious associations (Flint-Garcia et al. 2003; Würschum 2012). Several linear and mixed models have been proposed to correct for the effect of both confounding factors (Price et al. 2006; Pritchard et al. 2000; Yu et al. 2006) but the best fitting model will depend on the genetic architecture of the traits and their correlations with population and family structure. In pale flax, the *K* and *Q* + *K* models exhibited the best *P* value fitting for SPB and PH, although for most of the traits the three MLMs assessed performed similarly. In cultivated flax, however, the *K* and *Q* + *K* models tended to generate more false positives as compared to the PCA + *K* model, suggesting strong differences in selective and demographic forces shaping allele frequencies and trait architecture in both species. In pale flax, natural selection may have been the main force determining allele frequencies and genetic architecture of traits as an adaptive process, perhaps minimizing the negative effects of population structure on model performance for the traits assessed. On the contrary, the genotypes of the flax core collection have undergone divergent artificial selection for fiber, agronomic and seed quality traits, creating strong allele frequency differences.

### Marker–trait associations

The aim of the AM analysis was to identify genomic regions in pale flax potentially useful for cultivated flax breeding rather than identifying environment-specific QTL or QTL-by-environment interaction patterns. Significant associations were identified for seven and four traits in pale and cultivated flax, respectively, but none of the associated markers were common. These differences could be accounted by several factors including (1) the downside of unbalanced allele frequencies between flax morphotypes (Soto-Cerda et al. 2014), (2) differences in LD decay between pale and cultivated flax which requires lower marker density in the former to cover more LD blocks across the genome (Würschum 2012), and (3) differences in trait architecture and heritability (Würschum 2012). Despite the few marker-trait associations identified herein, we provided a proof of concept for pale flax functional variants as potentially useful for cultivated flax improvement.

Plant breeders have long recognized the value of wild relatives for the improvement of simply inherited traits, including disease and insect resistance or cytoplasmic male sterility (Feuillet et al. 2008; Xiao et al. 1998). Despite these successes, their uses in quantitative trait breeding such as yield have been rather limited because agriculturally desirable alleles are present in low frequency and are often masked by the effects of deleterious alleles in wild species (Swamy and Sarla 2008). Nevertheless, successful introgressions of exotic alleles to enhance yield have been reported in cultivated tomato (de Vicente and Tanksley 1993), cultivated oat, wheat, barley and maize (Frey et al. 1983). In pale flax, we identified associations with positive effects for TSW and SPB, two important yield components in flax breeding. Although the overall TSW and SPB values were lower than cultivated flax, pale flax’s alleles can improve the traits as demonstrated in cultivated tomato where de Vicente and Tanksley (1993) identified QTL for eleven traits that had an allelic effect opposite of what would be expected based on the poor phenotype of the wild parent. Similar observations were made in barley relatives (Schmalenbach et al. 2009). Recently, AM studies have identified favorable QTL for phenological, agronomical and kernel composition traits in teosinte (Weber et al. 2009), for spot blotch resistance in wild barley (Roy et al. 2010) and for fruit quality traits in wild tomato (Xu et al. 2013). Taken together, these studies demonstrate the potential to improve multiple traits in cultivated crops using wild relatives and AM.

Yield improvement through yield components and related traits such as flowering time and PH could be advantageous because of their simpler genetic architecture and higher stability than yield per se (Peng et al. 2011). PH is an important developmental and yield-related trait, and many genes regulating PH have been shown to affect harvest index and yield in rice (Xue et al. 2008), and yield and flowering time in soybean (Liu et al. 2011). Pale flax possessed five, six, nine and one novel alleles for TSW, SPB, PH and FL5 %, respectively, potentially useful for yield improvement through yield components in cultivated flax. For example, the favorable alleles associated with SPB and PH in pale flax, which were absent in cultivated flax, could be the first targets for the introgression of new functional variation in cultivated flax.

Flower color is determined by various pigments and co-pigments (Grotewold 2006). Flower color protects against UV-light and attracts pollinators, and the evolution of new pollinator associations are often accompanied by shifts in flower color (Fenster et al. 2004). In pale flax, marker Lu2725 explained ~97 % of PC variation, and based on the analysis of allele frequencies, the most common alleles in pale flax were absent in cultivated flax and vice versa. Comparison of phenotypic characters between both species revealed that most of the darker petal colors are absent in pale flax but they are in high frequency in cultivated flax, suggesting that PC could be another selected trait during domestication by ancient humans (Uysal et al. 2012).

Seed shattering, pod shattering or capsule dehiscence are common adaptive strategies in plants to ensure seed dispersal and survival. However, this commonly found wild species trait is undesirable in their domesticated counterparts because it results in yield losses prior to and during harvest (Lin et al. 2012). In pale flax, we identified markers Lu2344 and Lu442a associated with capsules dehiscence accounting for ~30 % of the variation. As with PC, the most common alleles in pale flax have been selected against in cultivated flax and vice versa. These markers could be exploited to perform early selection of non-dehiscent genotypes in interspecific populations, enabling removal of this undesirable trait while maintaining favorable exotic alleles.

### Allelic abundance and frequency of associated markers

Among the 12 associated markers identified herein, more than 48 % of the alleles were specific to pale flax, suggesting that these alleles most likely were lost through directional selection during domestication. The introgression of pale flax-specific alleles associated with TSW, SPB, PH and FL5% may be a useful strategy for widening the phenotypic variation in cultivated flax and counteract the inbreeding depression observed in the last decade or two in linseed in Canada. However, wild relatives’ introgressions are often associated with undesirable linkage drag (Feuillet et al. 2008). Depressed recombination between homeologous chromosomes is directly related to the evolutionary distance between species (Feuillet et al. 2008). Pale flax has been reported to cytogenetically differ from cultivated flax by a single translocation (Gill and Yermanos 1967), in agreement with morphological, phylogenetic and marker transferability evidence (Diederichsen and Hammer 1995; Fu and Allaby 2010; Fu and Peterson 2010; Uysal et al. 2010, 2012). Hence, recombination should not be an impediment to fully deploy pale flax in cultivated flax breeding. Current methods to transfer desirable wild alleles into cultivated material rely mostly on advanced backcross quantitative trait loci (AB-QTL) (Tanksley and Nelson 1996) and the introgression lines (ILs) methods (Zamir 2001). These approaches are limited to the evaluation of a single wild genotype at a time and require the need for validating the positive phenotypic effect of an exotic allele across different elite genetic backgrounds (Swamy and Sarla 2008). Alternatively, we propose the construction of interspecific MAGIC populations between pale and cultivated flax. This approach can overcome many of the limitations of conventional AB-QTL, ILs and AM populations (Morrell et al. 2012). For example, combining multiple parents across multiple generations of intermating can increase the rate of effective recombination per generation, improving genetic resolution, and the relatively even contribution of all parents to allele frequencies helps tackling the negative effects of population and family structures and is more effective in sampling rare alleles (Morrell et al. 2012).

In conclusion, our results corroborate the long held view that pale flax is a potential source of novel variation for cultivated flax. Pale flax contains not only high genetic diversity but also unique and rare alleles for agronomically important traits. To fully exploit this wild relative germplasm, new strategies such as MAGIC populations hold promise. However, the construction of a highly saturated linkage map is required for the accurate estimation of LD patterns and comparative mapping. The re-sequencing of the 125 pale flax accessions is underway and, along with the recently identified ~1.8 million single-nucleotide polymorphisms generated from the re-sequencing of the Canadian flax core collection, these resource promise to expand our knowledge of genome evolution, domestication events, LD patterns, QTL variation and architecture for multiple traits in cultivated flax and its wild ancestor, pale flax.

## Electronic supplementary material

Below is the link to the electronic supplementary material.

**Tab. S1** List of 125 pale flax accessions and their origin (DOCX 19 kb)

**Fig. S1** Number of populations (*K*) in pale and cultivated flax accessions using the *ad* *hoc* Δ*K* method (Evanno et al. 2005). **a** Estimation of *K* using the 532 pale and cultivated flax accessions. **b** Estimation of *K* within the 125 pale flax accessions. (TIFF 6128 kb)

**Tab. S2** AMOVA analysis of the five STRUCTURE populations (DOCX 12 kb)

**Fig. S2** Genome-wide linkage disequilibrium decay in pale flax. Scatter plot of LD decay (*r* ^2^) against the genetic distances (cM) for pairs of linked SSRs across the 15 linkage groups. The inner panel shows a detailed view of LD decay for markers located within 7 cM. The decay curves were plotted according to Breseghello and Sorrells (2006). The green line represents the threshold level of significance (*r* ^2^ = 0.1). The red line represents the average genome-wide LD of linked markers. Pairs of unlinked SSRs were assigned an arbitrary distance of 140 cM. (TIFF 7819 kb)

**Fig. S3** Comparisons of five association mapping models in pale and cultivated flax. P–P plots of observed versus expected −Log_10_ (*P*) values for six traits **a** Pale flax. **b** Cultivated flax. (TIFF 9098 kb)

**Fig. S4** Allelic abundance of twelve associated markers identified in pale flax and cultivated flax. The number of alleles including MAF < 0.05 identified in pale flax (red) and cultivated flax (blue) as well as the number of shared alleles (gray) is indicated. (TIFF 5140 kb)

**Fig. S5** Allelic abundance and frequency of markers associated with thousand seed weight, seeds per boll, plant height, start of flowering, petal color and capsular dehiscence in pale and their comparisons in cultivated flax. (TIFF 6773 kb)
