# Supplementary material for: The potential of pale flax as a source of useful genetic variation for cultivated flax revealed through molecular diversity and association analyses
Source: Mol Breed. 2014 Aug 12;34(4):2091–107. doi: 10.1007/s11032-014-0165-5 (PMC4544635; doi:10.1007/s11032-014-0165-5)
Supplement: Supplementary file 1 — Tab. S1 List of 125 pale flax accessions and their origin (DOCX 19 kb) [file 11032_2014_165_MOESM1_ESM.docx]

Table S1 List of 125 pale flax accessions and their origin

| **Accession nº** | **Origin** | **Accession nº** | **Origin** |
| --- | --- | --- | --- |
| LIN 5 | Unknown | LIN 1692 | France |
| LIN 11 | Unknown | LIN 1693 | France |
| LIN 1522 | Unknown | LIN 1694 | France |
| LIN 1525 | Unknown | LIN 1695 | France |
| LIN 1548 | Unknown | LIN 1696 | France |
| LIN 1555 | Unknown | LIN 1697 | Spain |
| LIN 1556 | Portugal | LIN 1698 | Italy |
| LIN 1601 | Spain | LIN 1699 | France |
| LIN 1602 | Spain | LIN 1700 | Italy |
| LIN 1606 | Spain | LIN 1701 | Italy |
| LIN 1607 | Spain | LIN 1702 | Italy |
| LIN 1610 | Italy | LIN 1703 | Italy |
| LIN 1623 | Italy | LIN 1704 | Georgia |
| LIN 1624 | Italy | LIN 1705 | Italy |
| LIN 1626 | France | LIN 1706 | Portugal |
| LIN 1627 | France | LIN 1708 | France |
| LIN 1628 | Italy | LIN 1709 | France |
| LIN 1629 | Italy | LIN 1710 | Ireland |
| LIN 1630 | France | LIN 1711 | United Kingdom |
| LIN 1631 | Italy | LIN 1745 | France |
| LIN 1641 | Italy | LIN 1746 | France |
| LIN 1642 | Italy | LIN 1747 | Italy |
| LIN 1643 | Spain | LIN 1748 | Italy |
| LIN 1644 | Italy | LIN 1749 | France |
| LIN 1670 | Georgia | LIN 1750 | France |
| LIN 1671 | Italy | LIN 1752 | Italy |
| LIN 1689 | Italy | LIN 1753 | France |
| LIN 1690 | France | LIN 1755 | France |
| LIN 1691 | France | LIN 1756 | Italy |
| LIN 1757 | Portugal | LIN 1854 | Italy |
| LIN 1768 | Italy | LIN 1855 | Portugal |
| LIN 1783 | Tunisia | LIN 1856 | France |
| LIN 1785 | France | LIN 1857 | Italy |
| LIN 1786 | Italy | LIN 1858 | Belgium |
| LIN 1788 | Italy | LIN 1859 | Italy |
| LIN 1798 | Unknown | LIN 1880 | France |
| LIN 1799 | Portugal | LIN 1881 | Unknown |
| LIN 1800 | Italy | LIN 1882 | France |
| LIN 1801 | France | LIN 1898 | France |
| LIN 1802 | Italy | LIN 1899 | Italy |
| LIN 1804 | France | LIN 1900 | Portugal |

Table S1 Continued

| **Accession nº** | **Origin** | **Accession nº** | **Origin** |
| --- | --- | --- | --- |
| LIN 1808 | Portugal | LIN 1909 | Portugal |
| LIN 1810 | France | LIN 1910 | Portugal |
| LIN 1811 | Italy | LIN 1911 | Portugal |
| LIN 1812 | Italy | LIN 1912 | Italy |
| LIN 1813 | France | LIN 1916 | Cyprus |
| LIN 1814 | Portugal | LIN 1917 | Spain |
| LIN 1815 | France | LIN 1918 | Spain |
| LIN 1816 | Italy | LIN 1922 | Italy |
| LIN 1843 | Unknown | LIN 1923 | Spain |
| LIN 1844 | Portugal | LIN 1924 | Spain |
| LIN 1845 | Portugal | LIN 1925 | Spain |
| LIN 1846 | Portugal | LIN 1926 | Spain |
| LIN 1847 | Portugal | LIN 1927 | Spain |
| LIN 1848 | Unknown | LIN 1928 | Spain |
| LIN 1849 | France | LIN 1929 | Spain |
| LIN 1850 | Portugal | LIN 1998 | Spain |
| LIN 1851 | Portugal | LIN 2005 | France |
| AMES 19348 | Portugal | PI 522291 | USA |
| PI 231886 | Belgium | PI 522292 | USA |
| PI 253971 | Iraq | PI 522307 | USA |
| PI 254371 | India | PI 522309 | USA |
| PI 511177 | USA |  |  |
