# Supplementary figures and images for: The potential of pale flax as a source of useful genetic variation for cultivated flax revealed through molecular diversity and association analyses
Source: Mol Breed. 2014 Aug 12;34(4):2091–107. doi: 10.1007/s11032-014-0165-5 (PMC4544635; doi:10.1007/s11032-014-0165-5)

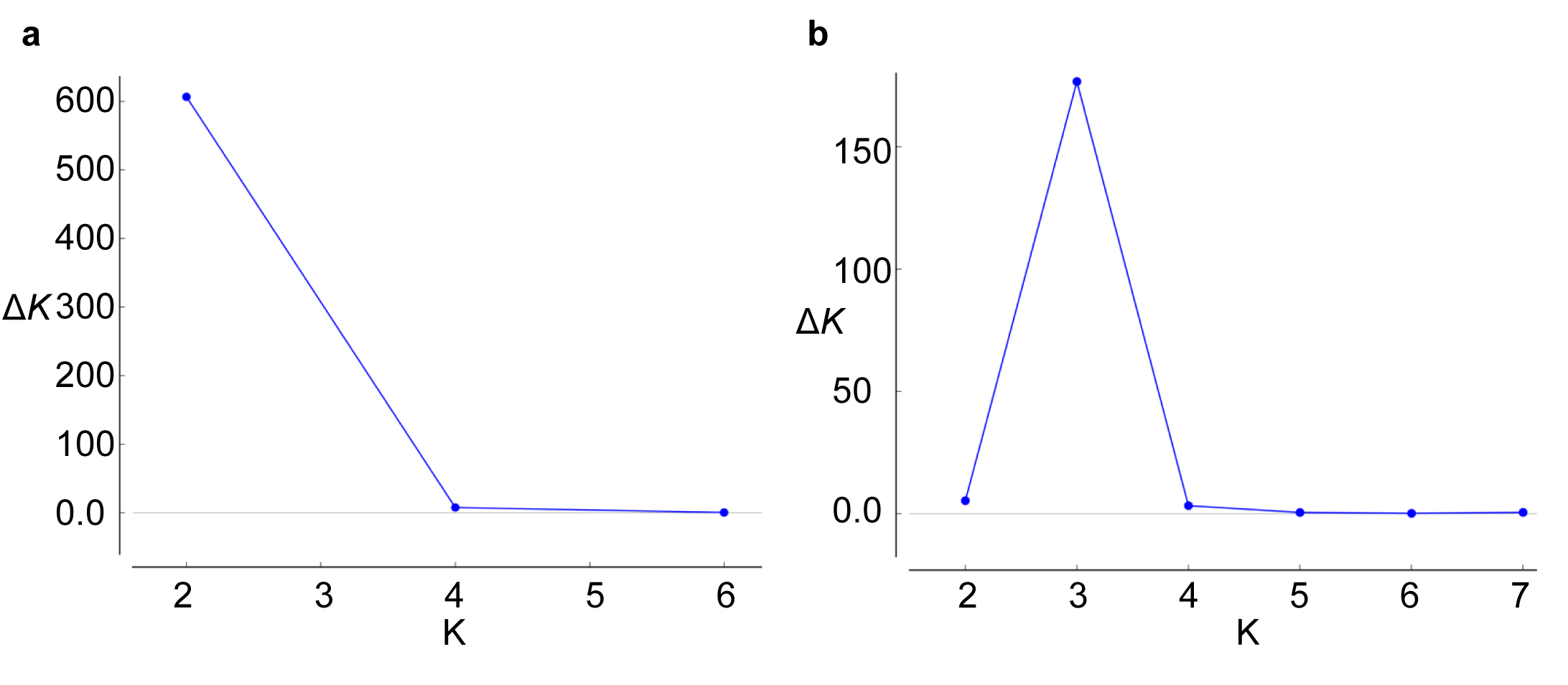

Supplement: Supplementary file 2 — Fig. S1 Number of populations (K) in pale and cultivated flax accessions using the ad hoc ΔK method (Evanno et al. 2005). a Estimation of K using the 532 pale and cultivated flax accessions. b Estimation of K within the 125 pale flax accessions. (TIFF 6128 kb) [file 11032_2014_165_MOESM2_ESM.tif]

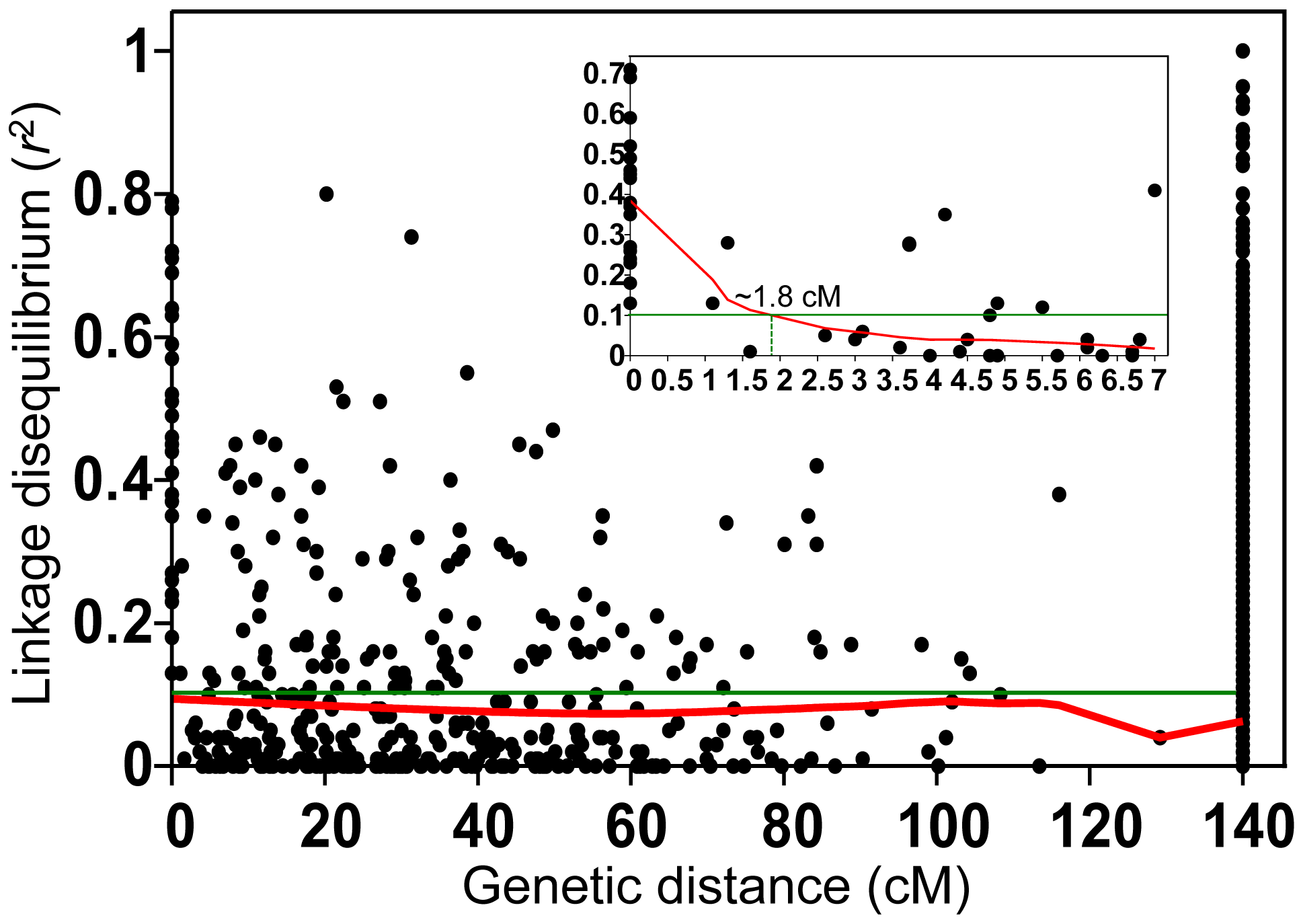

Supplement: Supplementary file 4 — Fig. S2 Genome-wide linkage disequilibrium decay in pale flax. Scatter plot of LD decay (r 2) against the genetic distances (cM) for pairs of linked SSRs across the 15 linkage groups. The inner panel shows a detailed view of LD decay for markers located within 7 cM. The decay curves were plotted according to Breseghello and Sorrells (2006). The green line represents the threshold level of significance (r 2 = 0.1). The red line represents the average genome-wide LD of linked markers. Pairs of unlinked SSRs were assigned an arbitrary distance of 140 cM. (TIFF 7819 kb) [file 11032_2014_165_MOESM4_ESM.tif]

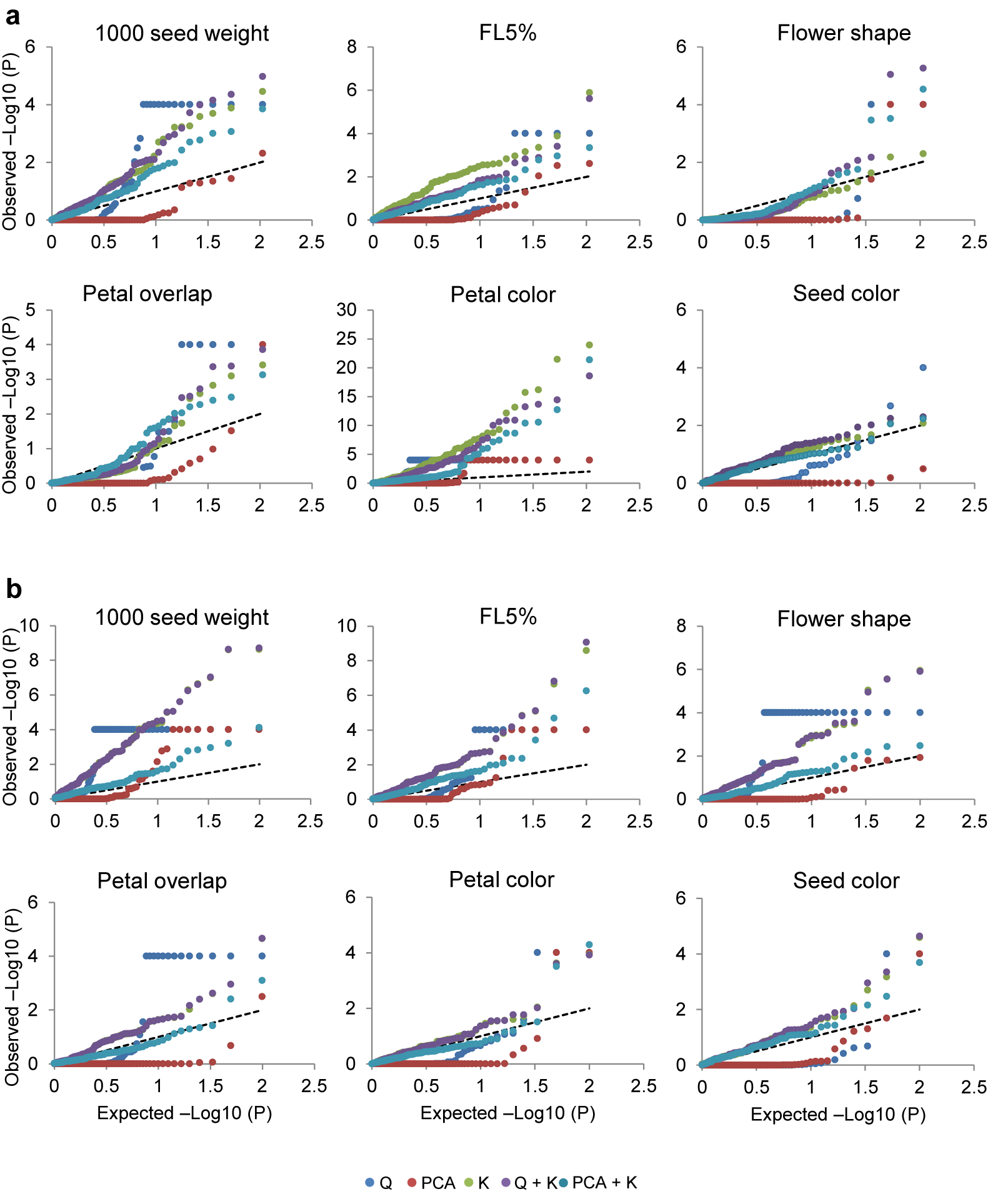

Supplement: Supplementary file 5 — Fig. S3 Comparisons of five association mapping models in pale and cultivated flax. P–P plots of observed versus expected −Log10 (P) values for six traits a Pale flax. b Cultivated flax. (TIFF 9098 kb) [file 11032_2014_165_MOESM5_ESM.tif]

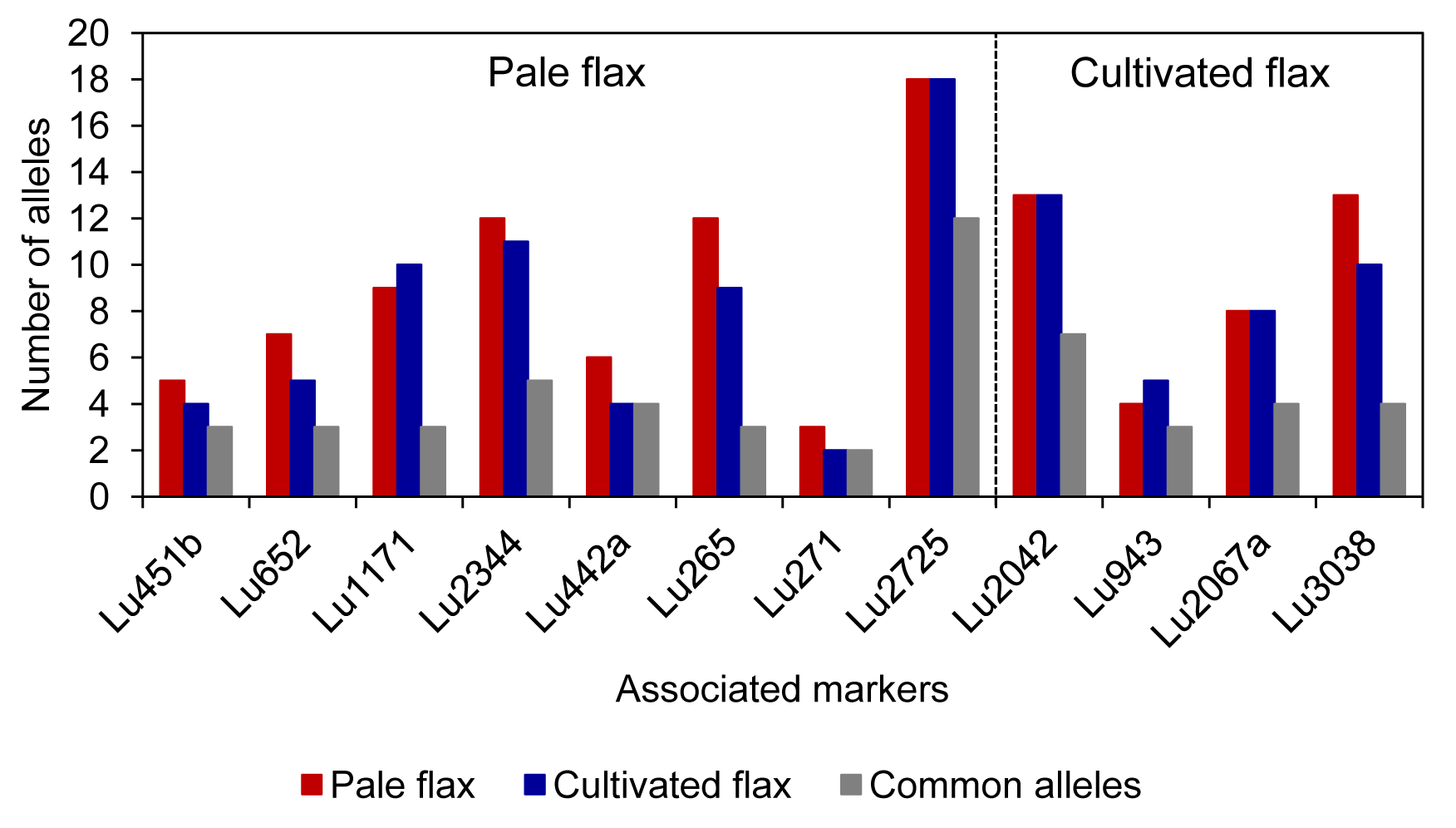

Supplement: Supplementary file 6 — Fig. S4 Allelic abundance of twelve associated markers identified in pale flax and cultivated flax. The number of alleles including MAF < 0.05 identified in pale flax (red) and cultivated flax (blue) as well as the number of shared alleles (gray) is indicated. (TIFF 5140 kb) [file 11032_2014_165_MOESM6_ESM.tif]

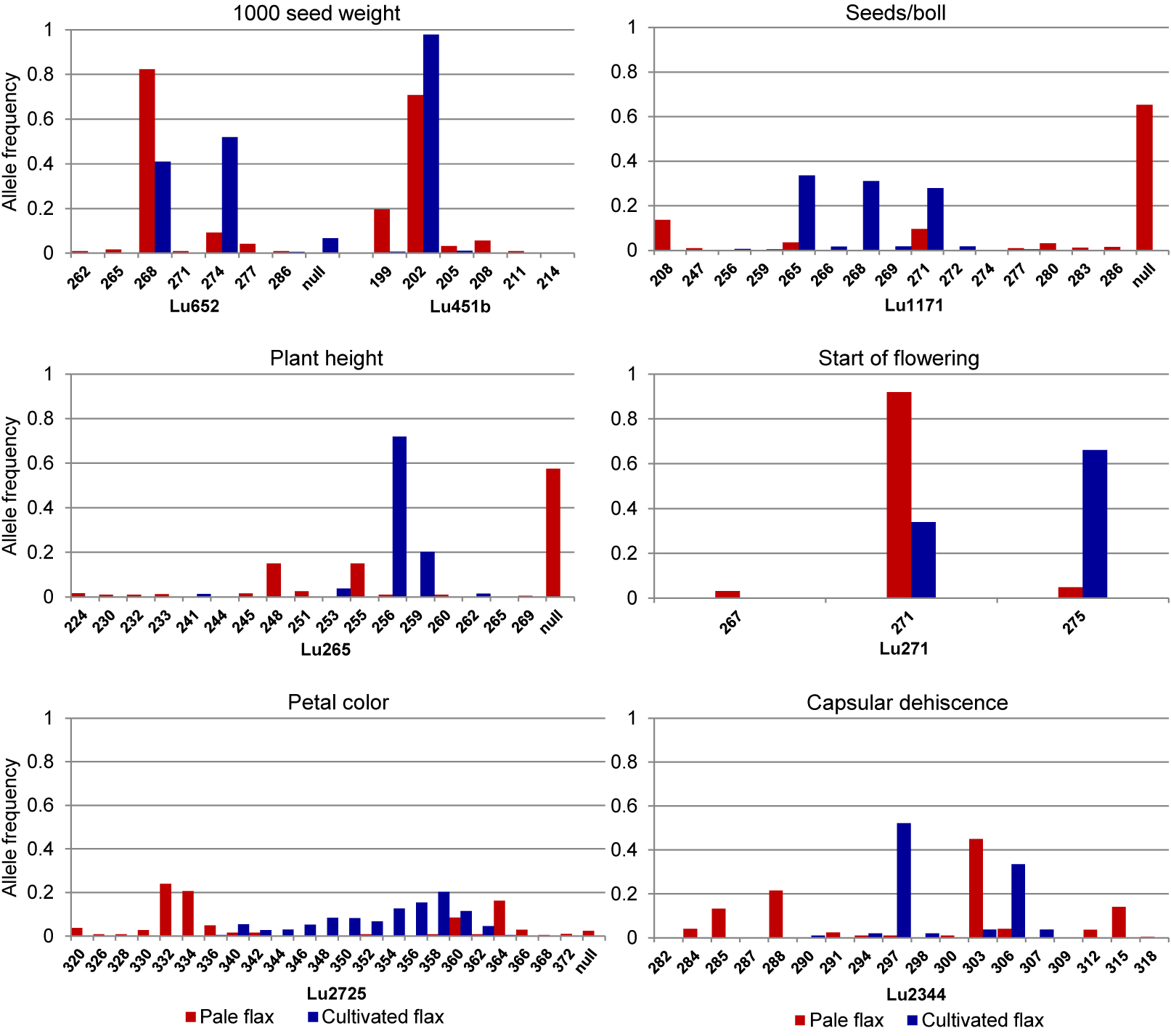

Supplement: Supplementary file 7 — Fig. S5 Allelic abundance and frequency of markers associated with thousand seed weight, seeds per boll, plant height, start of flowering, petal color and capsular dehiscence in pale and their comparisons in cultivated flax. (TIFF 6773 kb) [file 11032_2014_165_MOESM7_ESM.tif]
