# Supplementary material for: The potential of pale flax as a source of useful genetic variation for cultivated flax revealed through molecular diversity and association analyses
Source: Mol Breed. 2014 Aug 12;34(4):2091–107. doi: 10.1007/s11032-014-0165-5 (PMC4544635; doi:10.1007/s11032-014-0165-5)
Supplement: Supplementary file 3 — Tab. S2 AMOVA analysis of the five STRUCTURE populations (DOCX 12 kb) [file 11032_2014_165_MOESM3_ESM.docx]

**Table S2** AMOVA analysis of the five STRUCTURE populations

| **Source of**  **variation** | **Sum of**  **squares** | **Variance**  **components** | **Percentage**  **variation** |
| --- | --- | --- | --- |
| Among groups | 4444.72 | 8.36 | 19.8 |
| Among populations  within groups | 4068.18 | 7.53 | 17.8 |
| Among individuals  within populations | 26202.13 | 25.00 | 59.2 |
| Within individuals | 690.0 | 1.33 | 3.16 |
| Total | 35405.03 | 42.23 |  |
